# Editorial: Hereditary Spastic Paraplegias: At the Crossroads of Molecular Pathways and Clinical Options

**DOI:** 10.3389/fnins.2021.708642

**Published:** 2021-06-22

**Authors:** Andrea Martinuzzi, Craig Blackstone, Cahir J. O'Kane, Giovanni Stevanin

**Affiliations:** ^1^Department of Conegliano, Istituto di Ricovero e Cura a Carattere Scientifico “E. Medea” Scientific Institute, Pieve di Soligo, Italy; ^2^Department of Neurology, Massachusetts General Hospital, Boston, MA, United States; ^3^Department of Genetics, University of Cambridge, Cambridge, United Kingdom; ^4^Paris Sciences Lettres Research University, EPHE, Institut du Cerveau — Paris Brain Institute, Sorbonne Université, INSERM, CNRS, APHP, Paris, France

**Keywords:** hereditary spastic paraplegia, models, assessment, treatment, pathophysiology

Hereditary spastic paraplegias (HSPs) are a large group of rare neurologic conditions primarily affecting the longest corticospinal axons, with the pathognomonic triad of lower limb spasticity, weakness, and mild deep sensation impairment. Additional signs and symptoms are found in patients with complex (or complicated) forms. In spite of its low incidence and prevalence, the condition has attracted rapidly growing interest from the scientific community.

Why is a rare disorder initially described in the late nineteenth century by the German neurologist Adolph Strümpell and barely covered by the scientific literature throughout most of the twentieth century now exploding in terms of clinical, physiological, molecular and genetic interest? Certainly, the advent of next generation sequencing and the rapid identification of new disease genes play key roles. Indeed, the number of HSP disease loci is fast approaching one hundred. The paradox of extreme genetic heterogeneity, still growing (~40% of clinically diagnosed patients still escape molecular characterization) in the face of a mostly stereotypical clinical presentation, garners broad interest. Added to this is the conundrum of reconciling the multiple diverse cellular pathogenic themes proposed, which has piqued the interest of both basic and clinical scientists. It is thus not surprising that HSPs are among the conditions to which the large European initiative “Solve rare diseases” (http://solve-rd.eu/) has directed its attention, and there are also global efforts to gather data from dispersed clinical studies into structured, shared databases to facilitate investigations. In this issue of *Frontiers in Neuroscience*, 11 leading research groups from throughout the world discuss a diverse range of HSP topics, ranging from cellular and animal models to clinical investigations, emphasizing links of different cellular pathogenic themes to observable phenotypic and clinical manifestations.

The underlying physiopathologies of HSP dovetail with mechanisms currently in the spotlight for other, more common, neurologic disorders. These include the dysregulation of lipid metabolism, reviewed by Darios et al. in this issue and illustrated in detail by Sunderhaus et al. for PNPLA6 (SPG39). Lipid homeostasis is important for membrane maintenance and trafficking as well as synaptic and organelle functions, and alterations in the synthesis or degradation of various classes of lipids can led to CNS pathologies, which is not surprising for a tissue so highly enriched in lipids. Alterations of mitochondrial function, oxidative stress or impairment of axonal transport can occur in some HSP forms directly due to lipid metabolism impairment. On the other hand, lipid alterations can be observed in HSP forms associated to mutations in genes apparently unrelated to lipid metabolism, and the link between alterations of lipid metabolism and neurodegeneration remains unclear for many forms of HSP.

Endolysosomal functions are also commonly implicated in HSP; and Allison et al. show here that some variants in the MIT domain of the spastin protein do not directly affect its microtubule severing activity, as demonstrated for most other mutations, but rather alter endosomal tubule fission, molecule sorting via the tubular-vesicular pathway and lysosomal morphology, prefiguring future personalized “mutation-oriented” therapies. In addition, most neurodegenerative diseases have been considered for decades as resulting from cell autonomous mechanisms, meaning that single pathological events in neurons suffice to produce the disease. This has been reconsidered in many neurodegenerative conditions with the “neighborhood matters” concept, and HSP is no exception. Ozdowski et al. demonstrate here the important contributions of glial dysfunction to the synaptic defects in SPG4. They showed that the synaptic anomalies due to spastin knock-out can be reverted through Pak3 loss of function in glial cells; Pak3 is a kinase crucial for actin distribution and cell mobility, particularly important for glial projections at the neuromuscular junction.

While identification of human HSP causative genes has opened new routes to understanding the pathogenic mechanisms underlying these diseases, model organisms have proved invaluable in understanding how these genes, and mutations affecting them, affect neuronal function in whole organisms, from both genetic and physiological perspectives. Genetically, both Sunderhaus et al. and Montagna et al. use *Drosophila* to assay effects of disease-associated mutations in human HSP genes. Sunderhaus et al. show that HSP-causative alleles of human *PNPLA6* (*SPG39*) retain some biological functions in transgenic flies, and Montagna et al. provide evidence for loss-of-function effects of disease-causing mutations in *atlastin-1* (*SPG3A*). Transgenic targeted-expression tools were used by Ozdowski et al. to show the potential for glial cells to contribute to the phenotypes resulting from loss of spastin. Many HSP proteins are structural or enzymatic components of endoplasmic reticulum (ER) membrane, suggesting that axonal ER dysfunction might be a key mechanism for HSP. Thus, Oliva et al. and Napoli et al. characterize effects of HSP-related mutant genotypes on ER Ca^2+^ handling (Oliva et al.) and ER stress (Napoli et al.), and the latter study also shows pharmacological effects on the phenotypes. While not yet translatable to patients, the Napoli et al. study shows the potential of such models to assess pharmacological target proteins and processes. Finally, this collection contains two review articles that draw on genetic and physiological work on model organisms. Naef et al. review the genetic and cellular insights into complicated HSPs derived from zebrafish studies, while Fowler et al. review how interactions between organelles may be sites for some of the cellular defects that contribute to HSP pathology.

The clinical perspective is addressed from two important points of view: the search for clinically useful and efficient biomarkers (Montanaro et al.) and the evaluation of the efficacy of currently available interventions (Paparella et al.). The work on the multimodal MRI is particularly intriguing. Besides confirming the ability of so-called “advanced MRI” to capture widespread structural alterations in the brains of HSP patients, it provides the problematic finding of a paradoxical increase in Fractional Anisotropy (reflecting in certain ways the degree of order and organization of the axons within the central nervous tissue) in the longitudinal assessment of these patients. The interpretation of this finding is challenging. Some hypotheses are given in the paper, but certainly this is an area where information coming from studies on animal and cellular models might offer insight. The paper on the efficacy and safety of the approach combining chemodenervation with botulinum toxin and intensive physiotherapy highlights the importance of individualizing treatment solutions to the specific pattern of spasticity and motor impairment presented by each patient. In a condition characterized by wide heterogeneity of genes involved and pathways deranged in the setting of a shared principal phenotype (lower limb spasticity and weakness), this is a much needed reminder that the unifying feature of spastic paraplegia can mask subtle but clinically very relevant nuances that need to be taken into consideration when planning interventional trials. Again, a systematic appraisal of phenotypic patterns across the different SPG types might reveal patterns coherent with data emerging from the pathophysiology studies.

In summary, the goal of this scientific topic issue was to provide an arena for encounters between basic science advancements and clinical aspects in HSP. The topic has been addressed from various perspectives, but it is noteworthy that in addition to two papers overtly dedicated to the clinical approach, at least four other papers approaching the topic from a basic science perspective specifically mention the possible uses of these insights toward the development of much needed new therapies. This Research Topic issue in just a few months has attracted well over 30,000 views, confirming the fervent interest across the scientific and medical community for the HSPs. Indeed, the crossroads that the issue sought to portray between molecular pathways and clinical investigations looks more like a bridge, linking the two worlds.

## Author Contributions

All authors listed have made a substantial, direct and intellectual contribution to the work, and approved it for publication.

## Conflict of Interest

The authors declare that the research was conducted in the absence of any commercial or financial relationships that could be construed as a potential conflict of interest.

